# PIK3CA dependence and sensitivity to therapeutic targeting in urothelial carcinoma

**DOI:** 10.1186/s12885-016-2570-0

**Published:** 2016-07-28

**Authors:** R. L. Ross, H. R. McPherson, L. Kettlewell, S. D. Shnyder, C. D. Hurst, O. Alder, M. A. Knowles

**Affiliations:** 1 Section of Experimental Oncology, Leeds Institute of Cancer and Pathology, St James’s University Hospital, Beckett Street, Leeds, LS9 7TF UK; 2 Institute of Cancer Therapeutics, University of Bradford, Richmond Road, Bradford, BD7 1DP UK

**Keywords:** PIK3CA, PI3K signaling, Bladder cancer, Urothelium

## Abstract

**Background:**

Many urothelial carcinomas (UC) contain activating *PIK3CA* mutations. In telomerase-immortalized normal urothelial cells (TERT-NHUC), ectopic expression of mutant PIK3CA induces PI3K pathway activation, cell proliferation and cell migration. However, it is not clear whether advanced UC tumors are PIK3CA-dependent and whether PI3K pathway inhibition is a good therapeutic option in such cases.

**Methods:**

We used retrovirus-mediated delivery of shRNA to knock down mutant PIK3CA in UC cell lines and assessed effects on pathway activation, cell proliferation, migration and tumorigenicity. The effect of the class I PI3K inhibitor GDC-0941 was assessed in a panel of UC cell lines with a range of known molecular alterations in the PI3K pathway.

**Results:**

Specific knockdown of PIK3CA inhibited proliferation, migration, anchorage-independent growth and *in vivo* tumor growth of cells with *PIK3CA* mutations. Sensitivity to GDC-0941 was dependent on hotspot *PIK3CA* mutation status. Cells with rare *PIK3CA* mutations and co-occurring *TSC1* or *PTEN* mutations were less sensitive. Furthermore, downstream PI3K pathway alterations in *TSC1* or *PTEN* or co-occurring *AKT1* and RAS gene mutations were associated with GDC-0941 resistance.

**Conclusions:**

Mutant *PIK3CA* is a potent oncogenic driver in many UC cell lines and may represent a valuable therapeutic target in advanced bladder cancer.

**Electronic supplementary material:**

The online version of this article (doi:10.1186/s12885-016-2570-0) contains supplementary material, which is available to authorized users.

## Background

Advanced urothelial carcinoma (UC) of the bladder has a poor prognosis. At presentation, 15–30 % of UC patients are diagnosed with muscle-invasive tumors, and these carry a 5-year risk of death ranging from 33 to 73 % [1]. The standard treatment for localized invasive UC, is surgical removal of the bladder and regional lymph nodes, but metastatic disease is a major cause of death in these patients. The addition of cisplatin-containing combination neoadjuvant therapy has been shown to improve outcomes following cystectomy [2, 3], but metastasis remains common and although treatment with cisplatin-containing chemotherapy is beneficial in some cases, median survival for metastatic UC is only 13–15 months. As no significant improvements in survival have been achieved in recent years, new approaches to therapy, particularly second line therapies for metastatic disease, are urgently needed. Detailed molecular information on UC is now available [4, 5], but targeted agents have not yet been widely applied [6].

The phosphatidylinositol 3-kinase (PI3K) signaling pathway plays a critical role in regulation of cell metabolism, migration, proliferation and survival [7] and mutations that lead to aberrant activation of the pathway are found in virtually all types of cancer. In bladder cancer, 50–70 % of tumors contain mutations that are predicted to activate this pathway. These include activating mutations in *PIK3CA*, [8, 9] and *AKT1* [10], and inactivating mutations of *PTEN* [11, 12], *PIK3R1* [13], *TSC1* and *TSC2* [9, 14]. Assessment of the phosphorylation status of key pathway proteins confirms that pathway activation is present in bladder tumors of all grades and stages [15]. These tumors may benefit from PI3K-targeted therapy. Clinical trials of mTORC1 inhibitors in patients with bladder cancer have been initiated in recent years. In trials of the mTOR inhibitor Evirolimus, exceptional responses have been reported in patients with advanced UC whose tumors contained *TSC1* or *mTOR* mutations [16, 17]. In general however, responses to mTOR inhibitors have not been impressive [18], and indeed not all UC patients with tumors containing *TSC1* mutations have shown responses [16]. A potential reason is that mTOR inhibition triggers feedback loops that activate AKT [19]. Inhibitors of AKT have therefore been examined in preclinical studies of UC [20, 21]. Importantly, these studies revealed that sensitivity to AKT inhibition was strongly related to the presence of *PIK3CA* mutation. Taken together, it is clear that a thorough understanding of the signaling events initiated by the PI3K pathway is required in order to maximize clinical benefit.

Inhibition of PI3K as a potential therapeutic approach in UC has not previously been examined, though mutations in *PIK3CA* represent the most frequent PI3K pathway mutations in this cancer type, including 12–20 % of muscle-invasive tumors [14, 22]. Preclinical studies and early clinical trials indicate sensitivity to inhibitors of PI3K in several cancers including breast, ovarian, endometrial, lung and multiple myeloma [18, 23–29]. The majority of these studies highlight the Class 1 PI3K inhibitor, GDC-0941, as a good therapeutic drug for solid tumors. Furthermore, a phase I dose-escalation study of GDC-0941 has recently been completed and reports good tolerability of the drug with confirmed target modulation in tumor tissues [30]. Several studies in non-bladder cell lines have sought predictive biomarkers of sensitivity to PI3K inhibitors and it has been suggested that mutation of *PIK3CA* or loss of PTEN function are related to sensitivity to inhibitors of class I PI3K and that mutations in RAS genes are associated with resistance (Reviewed in [31]), though prediction based on these biomarkers is not absolute.

Previously we examined the effect of ectopic expression of mutant PIK3CA in telomerase-immortalized normal human urothelial cells (TERT-NHUC) and showed that this induces cell proliferation and migration [32]. In bladder tumors, more than one lesion in the PI3K pathway is commonly present [9] and this could potentially lead to distinct types of pathway dependence and response to specific therapeutic agents. Therefore, we have examined the consequences of specific inhibition of mutant PIK3CA in UC cells using stable knockdown, and treatment of a panel of UC cell lines containing a range of PI3K pathway alterations with the class I PI3K inhibitor, GDC-0941. Our findings strongly suggest that targeting of PIK3CA maybe a valid therapeutic approach in advanced bladder cancer.

## Methods

### Cell culture

Cell lines with known PI3K pathway mutation status were chosen (Additional file 1). Cell lines used for gene knockdown and functional studies were VM-CUB-3, BFTC909 and 253J. VM-CUB-3 was established from a primary human bladder transitional cell carcinoma (TCC), the grade and stage of which are unknown [33]. BFTC909 was established from the sarcomatoid component of a grade 3 TCC of the renal pelvis [34]. 253J was established from a retroperitoneal metastasis from a human TCC [35]. Bladder cancer cell lines J82, 253J, HT-1197, VM-CUB-3, BFTC909, UM-UC3, KU-19-19, DSH1, VM-CUB-1, CAL29, TCCSUP, MGH-U3, 639V, 97-1, LUCC1, LUCC3 and RT4 were used in drug sensitivity assays. Cell line identity was verified by short tandem repeat DNA typing using the Powerplex 16 kit (Promega). Profiles were compared to publically available data (ATCC, DSMZ) or where no reference profile was available, were confirmed as unique. Cells were grown in standard growth media; Hams F12 + 1 % FCS + 1 % Insulin-Transferrin-Selenium + 1 μg/ml hydrocortisone + 1x Non-essential amino acids + 2 mM L-glutamine (97-1), MEM + 10 % FCS + 1x Non-essential amino acids + 2 mM L-glutamine (HT-1197, J82, MGH-U3), DMEM + 10 % FCS + 2 mM L-glutamine (VM-CUB-3, VM-CUB-1, TCCSUP, BFTC909, 639V, CAL29, UM-UC3), McCoy’s 5a + 10 % FCS + 2 mM L-glutamine (RT4), 50:50 DMEM and RMPI 1640 + 5 % FCS + 2 mM L-glutamine (253J) and RPMI 1640 + 10 % FCS + 2 mM L-glutamine (DSH1, KU-19-19). Cells were incubated at 37 °C in 5 % CO_2_. TERT-NHUC [36] were also used and were cultured in Keratinocyte Growth Medium Kit 2 plus supplements (with 90 μl CaCl_2_). All cells were tested routinely for mycoplasma whilst in culture and before freezing by PCR using PCR Mycoplasma Test Kit I/C (PromoKine PK-CA91-1048) according to the manufacturer’s protocol.

### shRNA constructs and transduction of cell lines

Two shRNAs targeting PIK3CA were designed (forward oligo 1 5′- gcagaagtatactctgaaatTCAAGAGatttcagagtatacttctgcTTTTTTGGGCC-3′, reverse oligo 1 5′- CAAAAAAgcagaagtatactctgaaatCTCTTGAatttcagagtatacttctgc-3′, forward oligo 2 5′- caggtatctaccatggaggtTCAAGAGacctccatggtagatacctgTTTTTTGGGCC-3′ and reverse oligo 2 5′- CAAAAAAcaggtatctaccatggaggtCTCTTGAacctccatggtagatacctg-3′ according to an algorithm described previously [37, 38]. The sequence in lowercase is complementary to PIK3CA and forms a short-hairpin structure when expressed due to the intervening loop sequence. These, and a non-specific (NS) shRNA, were first cloned into pGEM-U6 puro and then into pRetroSuper-puro (pRS-puro), along with empty pRS-puro vector (control), to generate retroviruses to transduce VM-CUB-3, BFTC909 and 253J cell lines as described previously [37].

### Western blotting

Protein extraction was carried out as described [39] and concentration was quantified using the BIO-RAD protein assay (BIO-RAD, Hemel Hempstead, UK). SDS-PAGE and immunoblotting was carried out as described [32]. Primary antibodies were anti-p110a, anti-pAKT (Ser473), anti-panAKT (Cell Signaling) and anti-tubulin alpha (AbD Serotec). Bound primary antibodies were detected using HRP-conjugated secondary antibodies and Luminata Forte Western HRP Substrate (Millipore).

### Phenotypic assays

Proliferation, anchorage-independent growth, and Transwell migration assays were carried out and analyzed as described previously [32]. All assays were done in triplicate and repeated at least three times.

### Xenografts

Xenografts were established in mice by subcutaneous inoculation of VM-CUB-3 cells with PIK3CA knockdown and control cells (NS shRNA knockdown). Pure strain male BALB/cOlaHsd-Fox1^nu^ mice aged 6 to 8 weeks were used as described [40]. Each cell line was injected subcutaneously into sites on both flanks of 4 mice at a concentration of 1 × 10^7^ cells/site. On day 5 following inoculation, tumor was evident, and tumor volume was then measured frequently using calipers ((a^2^xb)/2; where a is the smaller and b is the larger diameter of the tumor) up to day 43. Tumor volume is shown in mm^3^.

### Immunohistochemistry

Tumors were formalin-fixed and embedded in paraffin wax. Sections were stained with haematoxylin and eosin, anti-human Ki-67 proliferation-associated antibody (Dako) and for apoptosis using the terminal deoxynucleotidyl transferase–mediated dUTP nick-end labelling (TUNEL) assay (ApopTag Plus Peroxidase *In Situ* Apoptosis Detection Kit; Chemicon) and analyzed as described previously [40].

### GDC-0941 drug treatment

The class I PI3K inhibitor, GDC-0941 (Axon Medchem), was used to treat bladder cancer cell lines and TERT-NHUC. The dose range chosen was based on previous studies that report IC_50_ values of 0.28–0.95 μM for cell viability of solid tumor cell lines, as well as pharmacokinetic data available from phase I clinical studies that report a maximum of 2 μM GDC-0941 plasma concentration in patients [30, 41]. Cell viability was assessed by CellTiter-Blue® (Promega) analysis of bladder cancer cell lines and TERT-NHUC subjected to GDC-concentrations from 0 to 2 μM. Cell viability was assessed by CellTiter-Blue® (Promega) analysis. 1000–4000 cells per well (number of cells determined to ensure that confluence was not achieved in untreated controls during the experiment) were plated in 96-well plates in five replicate wells and allowed to attach for 24 h before addition of 0–2 μM GDC-0941 in 0.1 % DMSO. After 72 h, 20 μl of CellTiter-Blue solution was added to the medium for 2 h and fluorescence read at 550 nm. Medium alone was used as a blank. Prism software (GraphPad Software, La Jolla, CA, USE) was used to calculate IC_50_ values.

Cell cycle and apoptosis analysis of cells cultured with 1 μM GDC-0941 or DMSO only for 48 h was evaluated by flow cytometry as described [40]. All assays were done in triplicate and repeated at least three times.

### Statistical analysis

Tumor growth was analysed using the Mann-Whitney U-test. Drug IC_50_ data was analyzed using the Fisher exact test (two-tailed, based on a cut off of >1 and <1 IC_50_ values) and Student’s t test was used to calculate significance of sensitivity of cells to GDC-0941 relative to PIK3CA wildtype and mutant status. Analysis of variance (ANOVA) and Student’s t-test (unpaired, two-tailed) were also used and *P* values were adjusted for multiple testing using the Bonferroni method. *P* < 0.05 was accepted as significant. All tests were conducted using Prism software, except for the Fisher exact test, which was conducted in R for Mac OS X 3.2.1.

## Results

### Knockdown of mutant PIK3CA in urothelial carcinoma cells reduces PI3K pathway signaling, and transformation-associated phenotypes

**Fig. 1:**
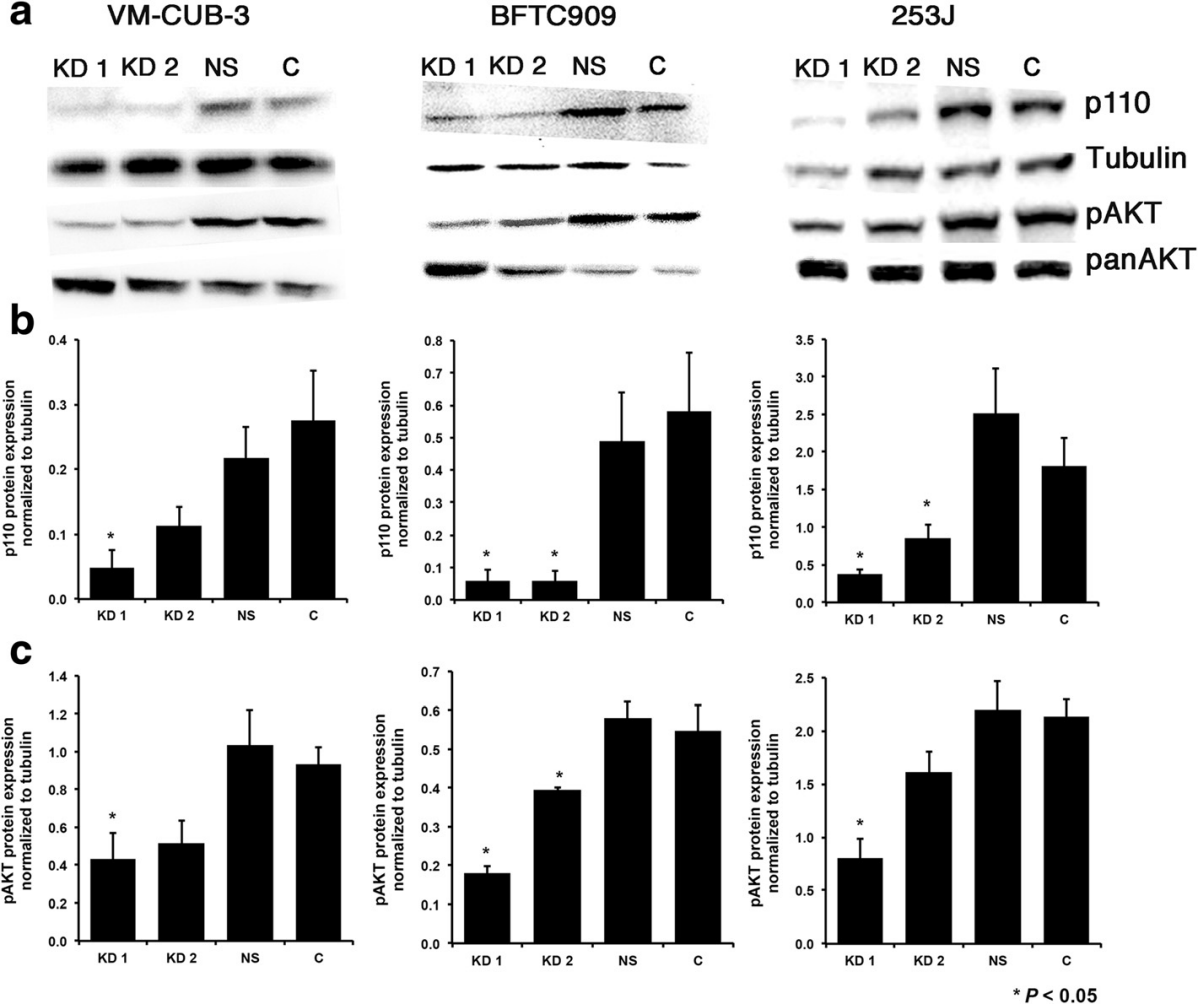
p110α and pAKT protein expression VM-CUB-3, BFTC909 and 253J cells with PIK3CA knockdown. **a**. Immunoblots showing p110**α** protein expression in cells transduced with shRNAs targeting PIK3CA (KD1 and KD2), non-specific control shRNA (NS) and control empty vector (C), and levels of phospho(p)AKT (Ser473) as read out of PI3K-AKT pathway activation. Cells were maintained in serum-supplemented conditions. **b**. Quantification of p110**α** expression relative to tubulin. **c**. Quantification of pAKT levels relative to total AKT. Results are representative of triplicate experiments. * indicates statistical significant difference from controls (Adjusted, *P <* 0.05) according to Student’s t test, adjusted using Bonferroni’s test for multiple comparisons. Values represent mean ± S.E

Two shRNAs targeting PIK3CA (KD1 and KD2) were designed and validated. These and a non-specific shRNA (NS) and empty vector (control) were retrovirally transduced into the UC cell lines VM-CUB-3, BFTC909 and 253J, all of which have mutations in codon E545 of *PIK3CA* (E545K in VM-CUB-3 and BFTC909; E545G in 253J), the most commonly mutated codon of *PIK3CA* in UC, and no known additional PI3K pathway aberrations (Additional file 1) [9, 10, 13, 42]. Following selection of mass populations of resistant cells, p110α protein levels were reduced by 42–92 % (average 69 %) in cells expressing each of the shRNAs (designated PIK3CA-KD) relative to controls and these levels remained constant through sequential cell passages, demonstrating efficient and stable PIK3CA knockdown (Fig. 1a & b).

Expression of E545K mutant PIK3CA in TERT-NHUC induces PI3K pathway activation and increases cell proliferation, migration and resistance to anoikis [32]. We tested whether these phenotypes are affected by knockdown of mutant PIK3CA in UC cell lines. In serum-supplemented medium, PIK3CA-KD cells showed significant reduction in phosphorylation of AKT (Ser473) levels relative to controls (33–73 %; mean 53 % reduction, Student’s t test; Adjusted *P* < 0.05). shRNA KD1 expression had the most profound effect in all 3 cell lines, compatible with it’s more significant effect on PIK3CA expression (Fig. 1a & c).

**Fig. 2:**
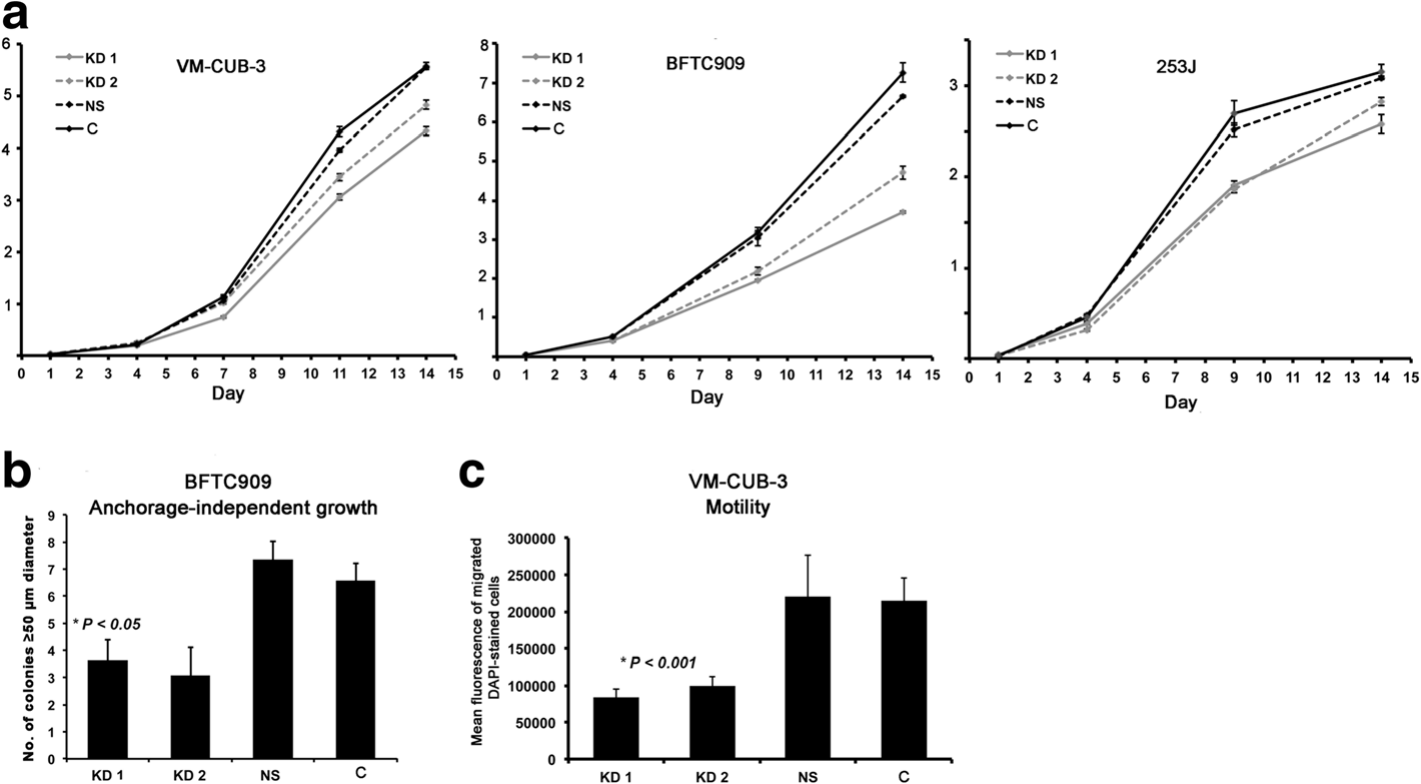
Proliferation, anchorage-independent growth and motility of bladder tumor cells following PIK3CA knockdown. Analysis of phenotypic consequences of PIK3CA knockdown. Bladder tumor cells with PIK3CA knockdown by shRNAs KD1 and KD2, non-specific control shRNA (NS) and vector control cells (C). **a**. Proliferation analysis. Cells were plated at 3x10^4^ cells per well in triplicate and counted on the days shown. Y axis is total cell number x 10^6^. Values represent mean ± S.E. Results are representative of triplicate experiments. **b**. Number of viable BFTC909 colonies with a diameter >50 μm in an area of 2.5 mm^2^ after 3 weeks of incubation in soft agarose. **c**. Mean fluorescence of migrated DAPI-stained VM-CUB-3 cells migrating through a Transwell membrane towards a chemoattractant (serum-supplemented medium). Values represent mean ± S.E. Results are representative of triplicate experiments. * indicates statistical significant difference (ANOVA test). Values represent mean ± S.E

PIK3CA-KD cells showed significantly reduced proliferation relative to controls (ANOVA test, *P* < 0.05), with cells expressing KD1 shRNA showing the greatest reduction (Fig. 2a). Annexin V cell staining showed no difference between PIK3CA-KD and control cells, indicating that apoptosis does not make a major contribution to the observed reduction in population growth (data not shown). Furthermore, treatment of PIK3CA-KD VM-CUB-3 cells with hydrogen peroxide in serum-supplemented and serum-depleted medium induced similar apoptotic indices to controls, indicating no change in sensitivity to apoptotic stimuli (data not shown).

Anchorage-independent growth was tested in BFTC909 cells, where a significant reduction in colony formation was found for PIK3CA-KD cells compared to controls, with statistical difference for KD1 cells (unpaired t test, *P* < 0.05) (Fig. 2b). VM-CUB-3 and 253J parental cells do not show measurable anchorage-independent colony formation. Cell migration through Transwell filters towards a chemoattractant (serum) was measured. PIK3CA-KD VM-CUB-3 cells showed a significant reduction in cell migration compared to controls (ANOVA test, *P* value < 0.001) (Fig. 2c). No significant differences in migration were observed between BFTC909-KD and 253J-KD cells and controls (data not shown). Taken together these results suggest that mutant PIK3CA drives increased proliferation in PIK3CA-mutant urothelial tumor cells, though other phenotypic consequences such as anchorage-independence and increased cell motility are not driven by PIK3CA alone. A summary of the PIK3CA-knockdown induced changes in cellular phenotypes in all the UC cell lines is illustrated in Additional file 2.

### Reduced tumorigenicity following knockdown of E545K PIK3CA

**Fig. 3:**
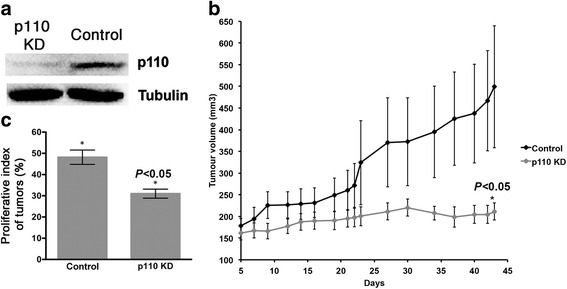
Growth of VM-CUB-3 cells with and without PIK3CA knockdown as xenografts. **a**. Immunoblot showing p110α protein expression in cells transduced with shRNA targeting PIK3CA (p110 KD) and non-specific control (control). **b**. 1 × 10^7^ PIK3CA knockdown cells and controls cells were injected into 2 sites each in 4 mice. Tumor volumes are shown in mm^3^. Difference in volumes of p110 KD and control tumors were statistically significant at 42 days of growth (Student t test, with using Bonferroni’s correction for multiple testing; P < 0.017) and overall after 43 days of growth (Mann-Whitney test, *P* < 0.05). **c**. Bar chart illustrates the percentage of Ki67 stained cells counted in 5 areas of each tumor section. * indicates statistical significant difference (ANOVA test, *P* 0.05). Values represent mean ± S.E

VM-CUB-3 cells can produce tumors *in vivo* in nude mice [43]. We tested the effect of knockdown of mutant PIK3CA expression on growth as subcutaneous xenografts. Independent transductions with the most potent shRNA (KD1) and NS shRNA generated p110α-KD and control cell lines respectively. Following selection of puromycin-resistant cell populations, p110α protein levels (Fig. 3a) and AKT activation (data not shown) were confirmed as described above. Cells were injected subcutaneously. Tumor volume (mm^3^) was measured from day 5 to day 43. p110α-KD xenografts showed significantly reduced growth rate compared with controls (Mann-Whitney test, *P* < 0.05) (Fig. 3b). Student t test showed that by day 42, the difference between tumor volume of KD and control xenografts was significantly different (Adjusted *P* < 0.017; on day 43 *P* < 0.005).

No significant differences were identified in tumor histology (data not shown) Sections were stained for Ki67 and TUNEL to assess effects on proliferation and apoptosis, respectively. A significant decrease in the proliferative index of p110α-KD tumors was found (ANOVA test, *P* < 0.05) (Fig. 3c and Additional file 3) but significant numbers of apoptotic cells were not detected, consistent with *in vitro* data.

### Effects of GDC-0941 on cell viability

The effects of mutant PIK3CA knockdown in UC cells suggest that this is a major driver of transformation in this cell type and that mutant PIK3CA is a good candidate for therapeutic targeting in UC. Therefore we assessed the effect of GDC-0941, a small molecule ATP-competitive inhibitor of class IA PI3K isoforms (α, β, δ), on a panel of UC cell lines and TERT-NHUC. We selected a panel of 17 UC cell lines with known mutation status for *PIK3CA, PIK3R1, AKT1, TSC1, PTEN* and the three RAS genes [9, 10, 13, 42] (Additional file 1). RNA expression data for *PIK3CA*, *PIK3CB* and *PIK3CD* were available for these and for normal human urothelial cells (Hurst and Knowles, unpublished data). In 13 of 17 lines, expression of *PIK3CA* was >2-fold higher than in normal urothelial cells. Six of these also showed >2-fold upregulation of *PIK3CB*. Expression of *PIK3CD* was >2-fold lower than in normal urothelial cells in all but two cell lines (Additional file 4).

**Fig. 4:**
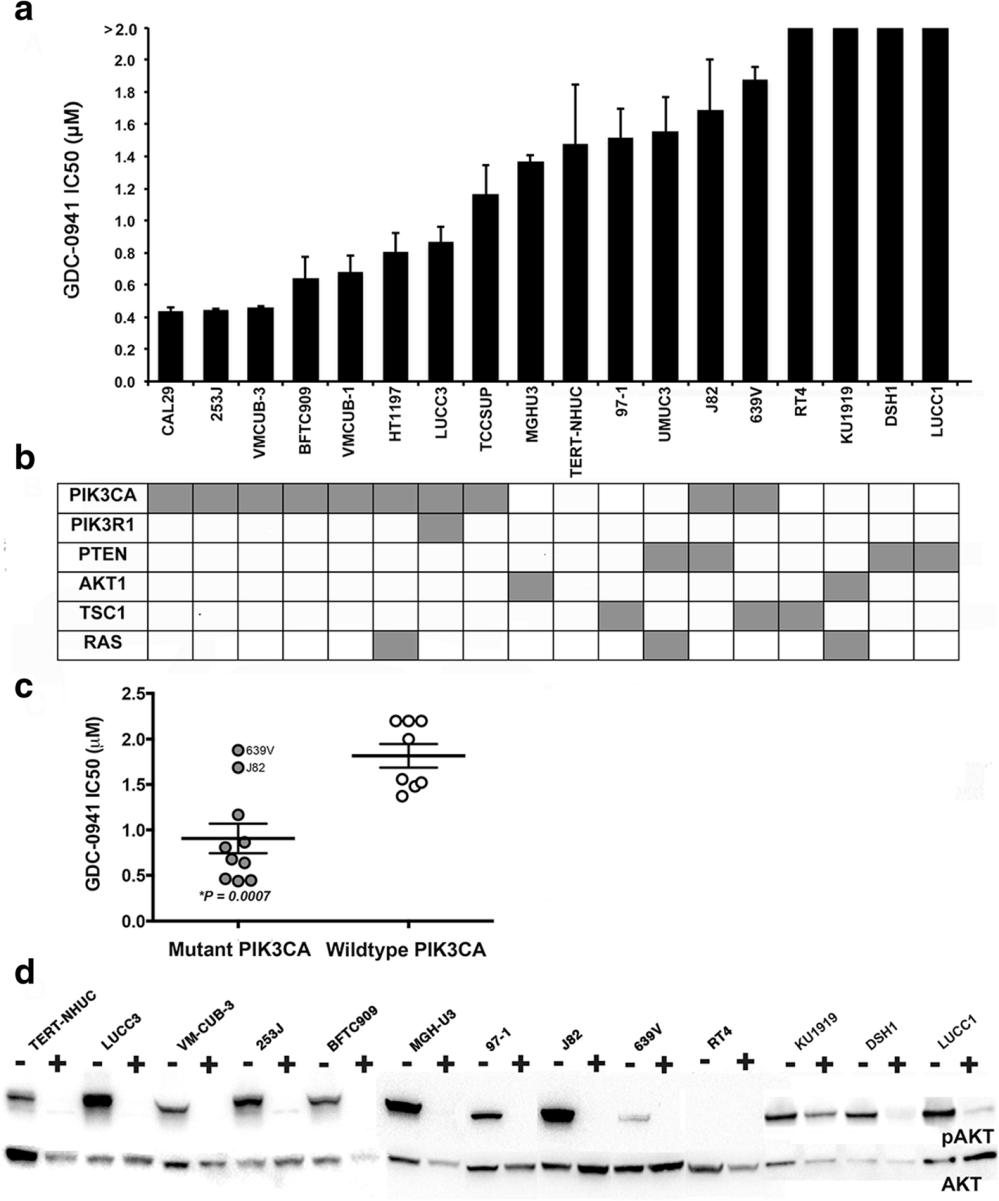
Activity of GDC-0941 in relation to PI3K pathway mutational status in bladder cancer cell lines. A panel of 17 bladder cancer cell lines and TERT-NHUC were treated with a range between 0–2 μM of GDC-0941 for 72 h and viability assessed. **a**. IC_50_ values shown as mean ± S.E, representative of triplicate experiments. **b**. Mutation status of cell lines. Grey represents presence of known mutation. **c**. Dot plot illustrating GDC-094-sensitive cell lines is linked to mutant *PIK3CA* status (grey and white circles represent mutant and WT *PIK3CA* cell lines, respectively). **d**. Immunoblot showing phospho-AKT (upper band) and total AKT (lower band) in cells treated with DMSO (−) or 1 μM GDC-0941 (+) for 1 h

Cells were cultured in 0-2.0 μM GDC-0941 for 72 h and cell viability measured relative to untreated controls. Sensitivity values (IC_50_) to this compound were within the same range as seen in other cancer cell types [25, 44]. Eight of the 10 cell lines with mutant *PIK3CA* showed IC_50_ values from 0.4 μM to 1.25 μM (Fig. 4 and Additional file 5). Six of these eight cell lines have only *PIK3CA* mutation and 2 harbor additional *PIK3R1* (LUCC3) or *NRAS* Q61R (HT-1197) mutations. Overall cell lines with wild-type *PIK3CA* were significantly less sensitive to GDC-0941 treatment than mutant *PIK3CA* cell lines (Fisher exact test, *P* < 0.05; two-tailed based on a cut off of >1 and <1 IC_50_ values). Figure 4c clearly illustrates that sensitivity to GDC-0941 is dependent on *PIK3CA* mutation status (according to an unpaired t test, *P* value 0.0007). The two cell lines with *PIK3CA* mutation but minimal response to the drug contained either homozygous deletion of *PTEN* (J82) or mutation of *TSC1* (639V). Furthermore, unlike the more GDC-0941-sensitive cell lines that harbor hotspot *PIK3CA* mutations, these two lines contain rare *PIK3CA* mutations (P124L and A1066V), which have been shown previously to be 5 times less potent than hotspot mutants in activating the PI3K pathway [32].

TERT-NHUC showed a higher IC_50_ (~1.5 μM) than cell lines with mutant *PIK3CA*. Three wildtype *PIK3CA* cell lines showed similar sensitivities to GDC-0941 treatment as TERT-NHUC. These cell lines harbored a single *AKT1* (MGH-U3) or *TSC1* (97-1) mutation or both *PTEN* and RAS mutations (UM-UC3). Four wildtype *PIK3CA* cell lines (RT4, KU19-19, DSH1 and LUCC1) showed varying degrees of GDC-0941 resistance (<10 % viability reduction at 2 μM), with RT4 (*TSC1* mutation) showing extreme resistance. Of these, DSH1 and LUCC1 have loss of PTEN, KU-19-19 has mutant *AKT1* and *NRAS* mutations. Therefore, downstream PI3K pathway alterations in *TSC1* or *PTEN* or co-occurring *AKT1* and RAS gene mutations were associated with GDC-0941 resistance.

Phosphorylation of AKT at Ser473 was examined in cell lines treated for 1 h with GDC-0941. Drug treatment reduced AKT phosphorylation to 0.5–7 % of that in untreated controls in all sensitive cell lines (Fig. 4d). Of the resistant cell lines, only LUCC1 (*PTEN* deleted) exhibited similar levels of pAKT reduction (3 %). Treatment of DSH1 (*PTEN* deleted) reduced AKT phosphorylation to 24 % of untreated levels. However, treatment of the resistant cell line KU-19-19 had little effect on AKT phosphorylation levels. This may be due to the presence of two mutations in *AKT1* (E17K and E49K), which was shown to increase AKT activity in comparison to the single mutant E17K found in the sensitive MGHU3 cell line [10]. Interestingly, no AKT phosphorylation was observable in RT4 under either untreated or GDC-0941-treated conditions, which is in agreement with previous studies [20].

**Fig. 5:**
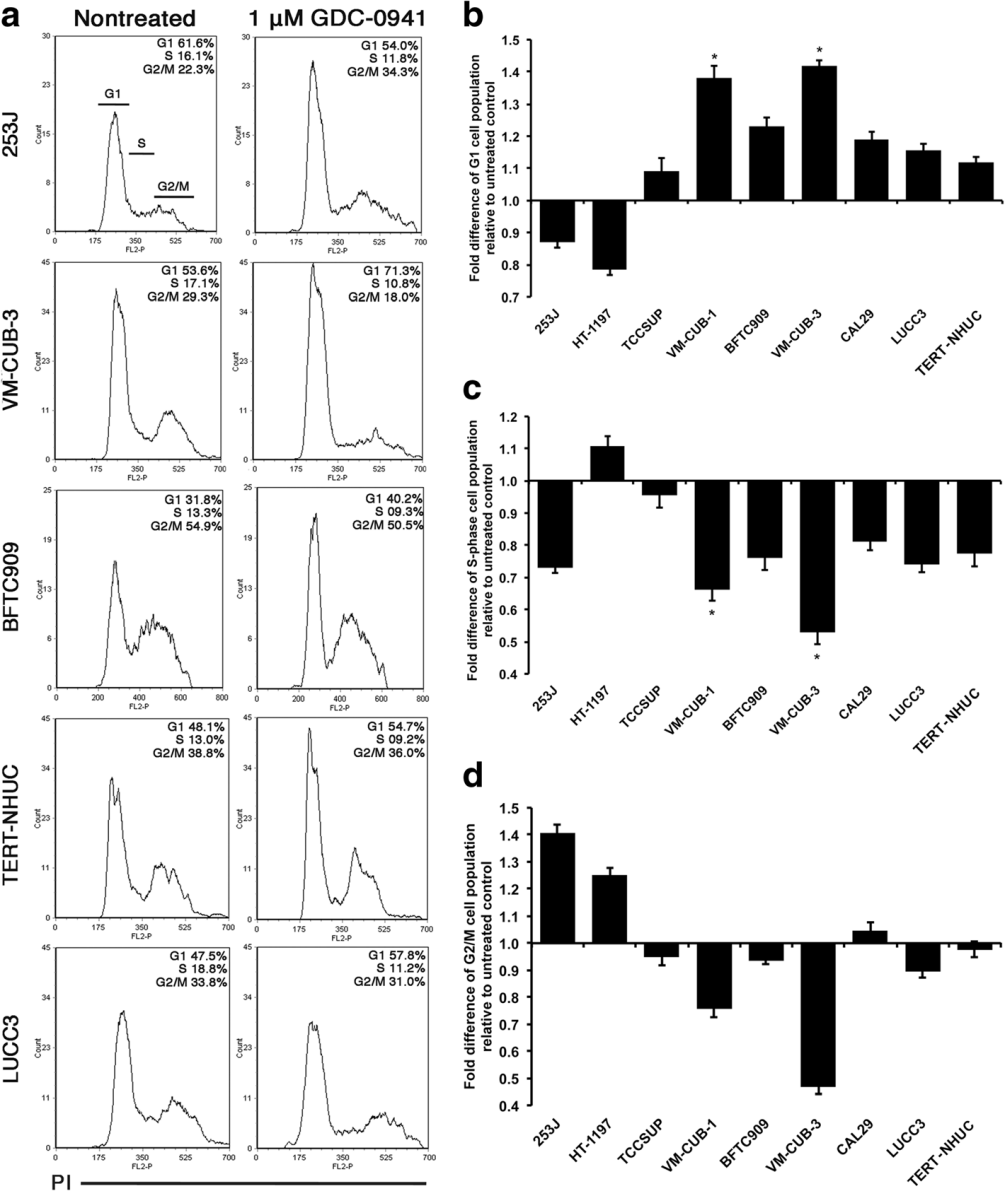
Flow cytometric analysis of cell cycle effects of GDC-0941 in bladder cancer cell lines and TERT-NHUC. **a**. Examples of FACS analysis of cells treated with DMSO vehicle (nontreated) or 1 μM GDC-0941 for 48 h. X-axis; DNA content assessed by propidium idodide staining and Y-axis; relative cell number. The legend to the right of each plot shows the percentage of cells in G1, S, and G2/M phase of the cell cycle. **b**-**d**. Quantification of replicate cell cycle assays as shown in (**a**, **b**). Fold difference of G1. **c**. Fold difference in S phase. **d**. Fold difference in G2/M. Values represent mean ± S.E and are representative of quadruplicate experiments. * indicates statistical significant difference (t test, *P* < 0.05)

**Fig. 6:**
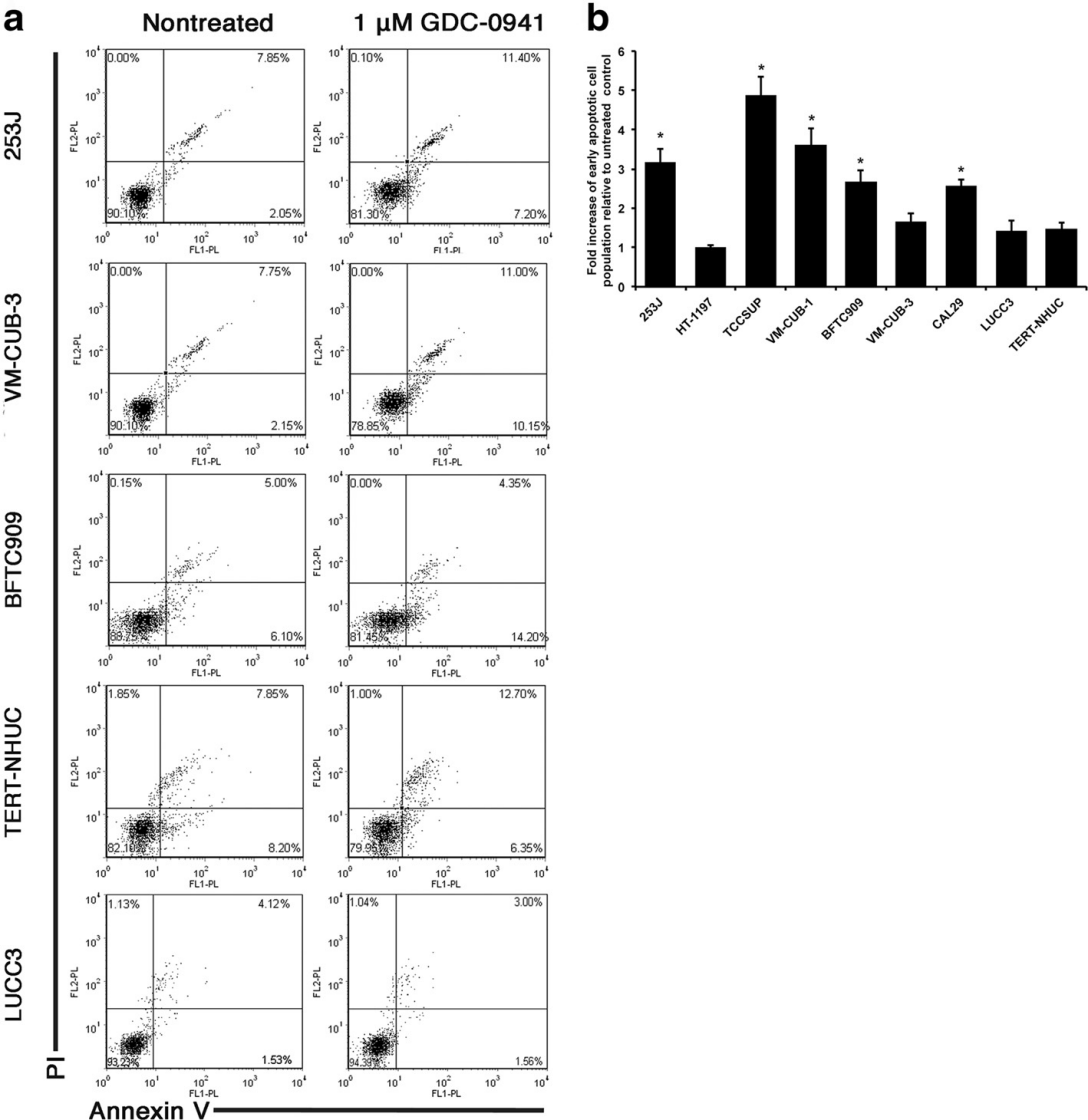
Flow cytometric analysis of apoptotic effects of GDC-0941 in bladder cancer cell lines and TERT-NHUC. **a**. Examples of FACS Annexin V/PI assay analysis of induction of apoptosis in cells treated with DMSO (nontreated) or 1 μM GDC-0941 for 48 h. X-axis, Annexin V; Y axis, Propidium Iodide (PI). Lower-right quadrant; Annexin V+ and PI- (early apoptotic cell population). **b**. Quantification of fold difference of early apoptotic cells in population treated with 1 μM GDC-0941 relative to nontreated cells from replicate assays to those shown in A. Values represent mean ± S.E and are representative of quadruplicate experiments. * indicates statistical significant difference (t test, *P* < 0.05)

To examine whether GDC-0941 treatment effects were cytostatic or cytotoxic, 8 sensitive cell lines with IC_50_ values up to 1.2 μM, and TERT-NHUC, were assessed for effects on cell cycle distribution and apoptosis. An increase in the proportion of cells in G_1_ accompanied by a decrease in S phase was observed in all cell lines except 253J and HT-1197 after 48 h of drug exposure, with the least effect in TCCSUP (Fig. 5a-c). However, only VM-CUB-1 and VM-CUB-3 showed statistically significant differences from TERT-NHUC (t test, *P* < 0.05). 253J and HT-1197 showed an increased fraction of cells in G2/M (Fig. 5d), which is consistent with the presence of a subset of larger cells. Five cell lines (253J, TCCSUP, VM-CUB-1, BFTC909 and CAL29) showed a significant increase in apoptotic index after 48 h of treatment relative to TERT-NHUC, with the greatest effect in TCCSUP (t test, *P* < 0.05) (Fig. 6a & b).

## Discussion

Activating mutations of *PIK3CA* are found in bladder tumors of all grades and stages. Whilst these are more common in tumors of low grade and stage (26–34 %) they are also found at significant frequency (12–20 %) in advanced UC (> stage T2) [8, 9, 22] (Hurst, Platt, and Knowles, unpublished data), which are commonly treated with systemic therapies and for which novel therapeutic approaches are urgently needed. Our objective was to examine the effects of specific inhibition of mutant PIK3CA in bladder tumor cells to determine whether mutant PIK3CA can be considered a valid therapeutic target in bladder cancer.

Stable knockdown of mutant E545K/E545G PIK3CA in three UC cell lines reduced PIK3CA protein levels by up to 92 % and was associated with reduced AKT activation, proliferation and *in vivo* tumor growth. This is consistent with observations reported in colon, gastric and ovarian cancer cell types [45–47], and with our previous data on the effects of expression of mutant PIK3CA in TERT-NHUC [32] and in other cell types [48–50]. Importantly, the intensity of reduction of these phenotypes was linked to the level of PIK3CA protein knockdown and the related reduction in AKT phosphorylation. We only observed inhibition of directional migration in VM-CUB-3 cells, which suggests that additional molecular alterations may contribute to this phenotype. It is possible that invasion may also be affected by PIK3CA and further investigation is warranted to investigate this. It is also important to note that the mice used for the *in vivo* work were immunodeficient. Whilst the *in vivo* results show a clear effect, and using immunodeficient mice allows the use of human tumor cells, there is a caveat in that interfering with the PI3K pathway can influence the immune system, and examination in a syngeneic model system could be relevant.

The class IA PI3K inhibitor GDC-0941 greatly reduced cell viability of UC cell lines with hotspot *PIK3CA* mutation status, similar to findings in other preclinical models [25–27, 41, 51]. The two cell lines with rare *PIK3CA* mutations had minimal response to the drug and contained either homozygous deletion of *PTEN* (J82) or mutation of *TSC1* (639V), suggesting that co-existing *PTEN* or *TSC1* mutations confer some resistance to PI3K inhibition. Cell lines with wildtype *PIK3CA* were less sensitive or lacked sensitivity to GDC-0941 completely, in agreement with studies in breast cancer, multiple myeloma, lung cancer, and endometrial cancer, where *PIK3CA* mutations are also frequent [25, 26, 51]. Interestingly, our data support the findings of a study that examined the effect of the pan-AKT inhibitor, MK-2206, on the viability of UC cell lines [20]. Hotspot mutant PIK3CA expressing cell lines, 253J, HT1197 and VMCUB-1 were sensitive to AKT inhibition, whereas cell lines with rare *PIK3CA* mutations (J82 and 639V) or those with *PTEN* and RAS gene or *TSC1* mutations (UMUC3 and RT4) had IC_50_ values above 2 μM (classed as resistant).

As reported in several other tumor types, additional information on PI3K pathway mutational status is needed to correctly predict the response to PI3K inhibition. There have been conflicting reports of the effect of *PTEN* mutation on sensitivity to class I PI3K inhibition. For example, in non-small cell lung carcinoma, endometrioid and breast carcinoma cell lines, PTEN loss of expression or mutation was associated with sensitivity to GDC-0941 [25, 26, 51] but in multiple myeloma cell lines [27], PTEN loss had no predictive value. In the present study, all UC lines with PTEN loss as a single alteration showed resistance. PI3K pathway-dependent cells with mutations in genes that act below PIK3CA in the pathway are predicted to be resistant to PI3K inhibition. Indeed, our data showed that *PTEN, AKT1* and *TSC1* mutant UC cell lines were less sensitive than those with *PIK3CA* mutations, and in the case for RT4 (*TSC1* mutant), resistant to PI3K inhibition.

Similarly, in some studies RAS mutation has been reported to predict resistance to PI3K inhibition and in others to have no impact on sensitivity [25, 52]. Here we found that HT-1197, which harbors both *PIK3CA* and *NRAS* mutations, was sensitive to GDC-0941 but other lines with RAS mutations co-occurring with *PTEN* or *AKT1* mutations were less sensitive. This is likely to be related to relative dependence on RAS-MAPK and PI3K pathways in individual cases. Recent observations have highlighted the effect of context-dependent crosstalk on MEK signaling associated with inhibition of PI3K in breast cancer [53, 54]. Interestingly, a phase I clinical trial of GDC-0941 in solid cancers showed that a melanoma patient with good response had a BRAF mutation and wild-type *PIK3CA* [30]. As many bladder cancers have alterations that are known to activate the MAPK pathway [4], it is likely that dual MEK and PI3K inhibition may be advantageous. Results from ongoing clinical trials of GDC-0941 in combination with drugs including GDC-0973 (MEK inhibitor), erlotinib (EGFR inhibitor), fulvestrant, and cisplatin are awaited.

In this study, shRNA knockdown of PIK3CA in three cell lines clearly demonstrated dependence on this p110 isoform. However, as many of the cell lines examined expressed PIK3CB, we cannot rule out that the effects of GDC-0941 on PIK3CB may have contributed to the observed effects of this inhibitor. As p110α-specific drugs e.g. NVP-BYL719 [55], are now in clinical trials, it will be important to assess the relative dependence of each isoform in UC, prior to consideration of potential clinical studies.

Levels of pAKT (Ser473) were decreased by GDC-0941 treatment, independent of whether a cell line was sensitive or resistant, as previously reported [26, 27, 56]. Resistant cell lines may not be dependent on AKT signaling or may have additional mechanisms to activate the PI3K pathway. Interestingly, RT4, the most resistant cell line in this study, had an undetectable basal level of AKT activation compared to the other cell lines studied. As this cell line has mutant *TSC1,* this implies that only the mTOR branch of the pathway is active in this case, as shown previously [20]. As many *PIK3CA* mutant cancers rely on effectors other than AKT, such as PDK1 and its substrate SGK3 [57], phospho-AKT may not be an ideal pharmacodynamic biomarker for relevant PI3K inhibition.

The effect of GDC-0941 in the majority of sensitive bladder cancer cells was via induction of both G1 cell cycle arrest and some apoptosis. Thus in some contexts, single agent GDC-0941 may exert cytotoxic and cytostatic effects in UC as seen in other tumor models [26, 27, 58]. Dual inhibition of mTOR and PI3K has been shown to be a promising approach in cell lines from other cancer types [29] and may be particularly efficacious in bladder where molecular lesions are found in multiple genes in the PI3K pathway, often concurrently [4].

## Conclusions

Our observations indicate a good therapeutic window for PI3K inhibitors in some bladder cancers that harbor PI3K hotspot mutations, as well as those with co-existing *NRAS* mutations. The first in human phase I clinical study of GDC-0941 in patients with advanced solid tumors has recently been completed and showed promising results with minimal toxicities at doses sufficient to decrease PI3K pathway activation [30]. In UC, a disease for which targeted therapy is still in early stages, our data suggest that PI3K targeted therapy can benefit patients with *PIK3CA* mutations and that mutation can act as a biomarker for patient selection. Additional preclinical work is warranted to assess the impact of dual mTOR-PI3K and MEK-PI3K inhibition on a large panel of UC cell lines with a range of PI3K/AKT and MEK/ERK pathway genetic alterations to fully understand the context for optimal therapy of PI3K pathway-dependent bladder tumors. Currently, trials of GDC-0941 in combination with other agents suggested by preclinical studies, such as paclitaxel and inhibitors of MEK1 and EGFR [59–61], are in progress.

## Abbreviations

KD, knockdown; NS, non-specific; PI, propidium iodide; PI3K, phosphatidylinositol 3-kinase; TCC, transitional cell carcinoma; TERT-NHUC, telomerase-immortalized normal urothelial cells; UC, urothelial carcinoma.

## Additional files

Additional file 1: PI3K pathway alterations in the panel of bladder cancer cell lines used in GDC-0941 viability assay. (DOCX 130 kb) Additional file 2: The relationship between knockdown of PIK3CA and characteristics in UC cell lines. (DOCX 72 kb) Additional file 3: Ki67 immunohistochemistry analysis of tumors generated from PIK3CA KD and controls (non-specific KD) xenografts. (TIF 4887 kb) Additional file 4: Expression of PIK3CA, PIK3CB and PIK3CD in bladder cancer cell lines measured by microarray. (TIF 370 kb) Additional file 5: The effect of GDC-0941 on viability of bladder cancer and TERT-NHUC cell lines. (TIF 701 kb)
