# Supplementary material for: PIK3CA dependence and sensitivity to therapeutic targeting in urothelial carcinoma
Source: BMC Cancer. 2016 Jul 28;16:553. doi: 10.1186/s12885-016-2570-0 (PMC4964013; doi:10.1186/s12885-016-2570-0)
Supplement: Additional file 1: — PI3K pathway alterations in the panel of bladder cancer cell lines used in GDC-0941 viability assay. (DOCX 130 kb) [file 12885_2016_2570_MOESM1_ESM.docx]

|  | *PIK3CA* | *PIK3R1* | *AKT* | *TSC1* | *PTEN* | *RAS* |
| --- | --- | --- | --- | --- | --- | --- |
| TERT-NHUC | WT | WT | WT | WT | WT | WT |
| 253J | E545G | WT | WT | WT | WT | WT |
| BFTC909 | E545K | WT | WT | WT | WT | WT |
| J82 | P124L | WT | WT | WT | HD | WT |
| HT-1197 | E545K | WT | WT | WT | WT | NRAS: Q61R |
| VM-CUB-3 | E545K | WT | WT | WT | WT | WT |
| CAL29 | H1047R | WT | WT | WT | WT | WT |
| TCCSUP | E545K | WT | WT | WT | WT | WT |
| MGH-U3 | WT | WT | E17K | WT | WT | WT |
| 639V | A1066V | WT | WT | F285V, H617D | WT | WT |
| 97-1 | WT | WT | WT | R692* | WT | WT |
| LUCC3 | E545K | E507Q, R557P | WT | WT | WT | WT |
| RT4 | WT | WT | WT | 1669delC | WT | WT |
| VM-CUB-1 | E545K | WT | WT | WT | WT | WT |
| UM-UC3 | WT | WT | WT | WT | HD | KRAS: G12C |
| KU-19-19 | WT | WT | E17K,  E49K | WT | WT | NRAS: Q61R |
| DSH1 | WT | WT | WT | WT | HD | WT |
| LUCC1 | WT | WT | WT | WT | HD | WT |

**Additional file 1.** PI3K pathway alterations in the panel of bladder cancer cell lines used in GDC-0941 viability assay[^1-4^](#_ENREF_1)

References

1 Askham JM, Platt F, Chambers PA, Snowden H, Taylor CF, Knowles MA (2010). AKT1 mutations in bladder cancer: identification of a novel oncogenic mutation that can co-operate with E17K. *Oncogene* **29:** 150-155.

2 Hurst CD, Zuiverloon TC, Hafner C, Zwarthoff EC, Knowles MA (2009). A SNaPshot assay for the rapid and simple detection of four common hotspot codon mutations in the PIK3CA gene. *BMC Res Notes* **2:** 66.

3 Platt FM, Hurst CD, Taylor CF, Gregory WM, Harnden P, Knowles MA (2009). Spectrum of phosphatidylinositol 3-kinase pathway gene alterations in bladder cancer. *Clin Cancer Res* **15:** 6008-6017.

4 Ross RL, Burns JE, Taylor CF, Mellor P, Anderson DH, Knowles MA (2013). Identification of Mutations in Distinct Regions of p85 Alpha in Urothelial Cancer. *PloS One* **8:** e84411.
