# Supplementary material for: PIK3CA dependence and sensitivity to therapeutic targeting in urothelial carcinoma
Source: BMC Cancer. 2016 Jul 28;16:553. doi: 10.1186/s12885-016-2570-0 (PMC4964013; doi:10.1186/s12885-016-2570-0)
Supplement: Additional file 2: — The relationship between knockdown of PIK3CA and characteristics in UC cell lines. (DOCX 72 kb) [file 12885_2016_2570_MOESM2_ESM.docx]

| **UC CELL LINE** | p110α protein levels | pAKT protein levels | Proliferation | Agar growth | Migration |
| --- | --- | --- | --- | --- | --- |
| VMCUB3 KD1 | + | + | + | - | + |
| KD2 | ++ | ++ | ++ | - | + |
| NS | +++ | +++ | +++ | - | +++ |
| CONTROL | +++ | +++ | +++ | - | +++ |
| BFTC909 KD1 | + | + | + | + | - |
| KD2 | + | ++ | ++ | + | - |
| NS | +++ | +++ | +++ | +++ | - |
| CONTROL | +++ | +++ | +++ | +++ | - |
| 253J KD1 | + | + | + | - | - |
| KD2 | ++ | ++ | ++ | - | - |
| NS | +++ | +++ | +++ | - | - |
| CONTROL | +++ | +++ | +++ | - | - |

**Additional file 2.** The relationship between knockdown of PIK3CA and characteristics in UC cell lines.

Symbols + represents the strength of the protein levels/phenotype, with +++ indicating the strongest affect and + the least affect.
