# Supplementary figures and images for: PIK3CA dependence and sensitivity to therapeutic targeting in urothelial carcinoma
Source: BMC Cancer. 2016 Jul 28;16:553. doi: 10.1186/s12885-016-2570-0 (PMC4964013; doi:10.1186/s12885-016-2570-0)

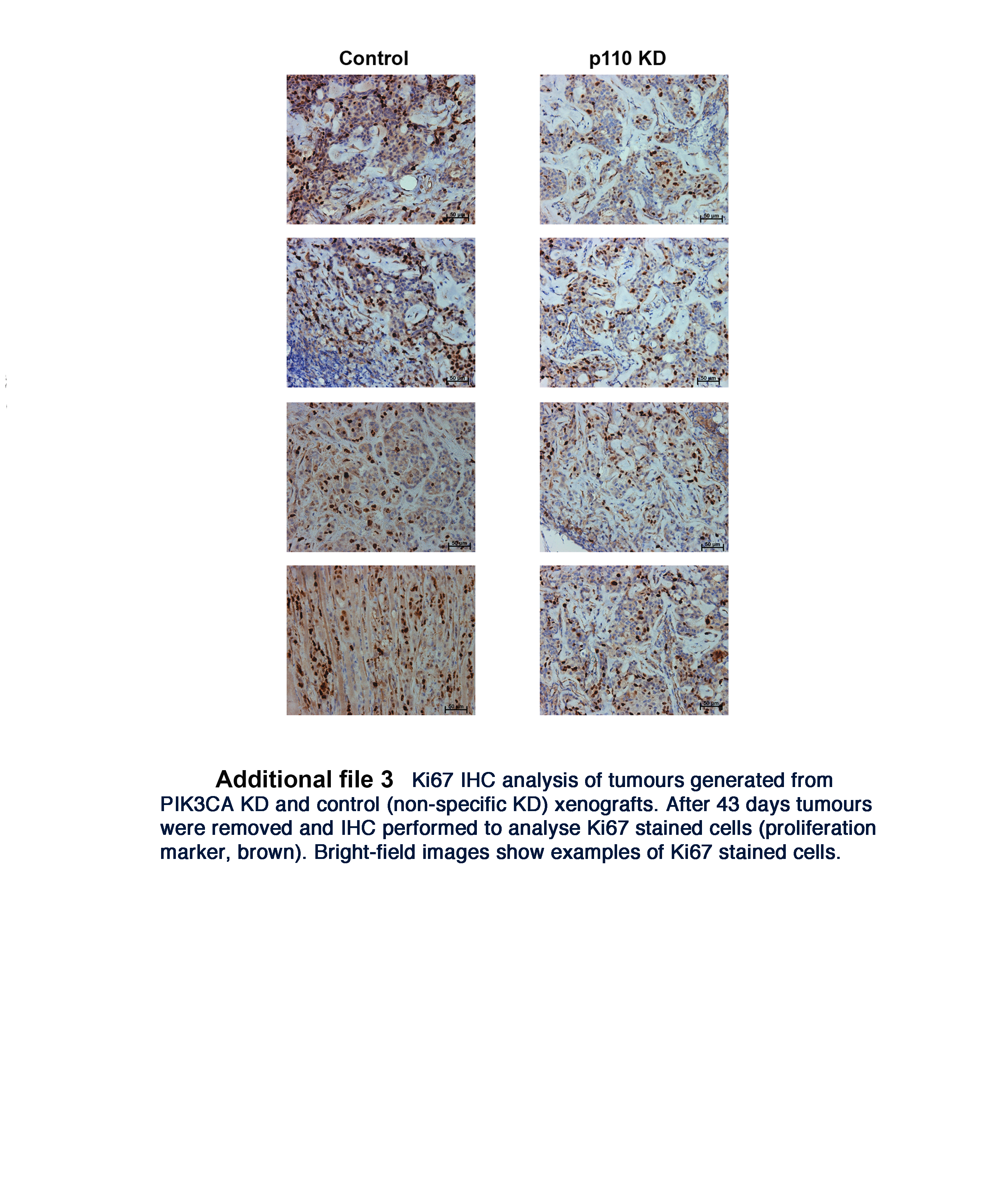

Supplement: Additional file 3: — Ki67 immunohistochemistry analysis of tumors generated from PIK3CA KD and controls (non-specific KD) xenografts. (TIF 4887 kb) [file 12885_2016_2570_MOESM3_ESM.tif]

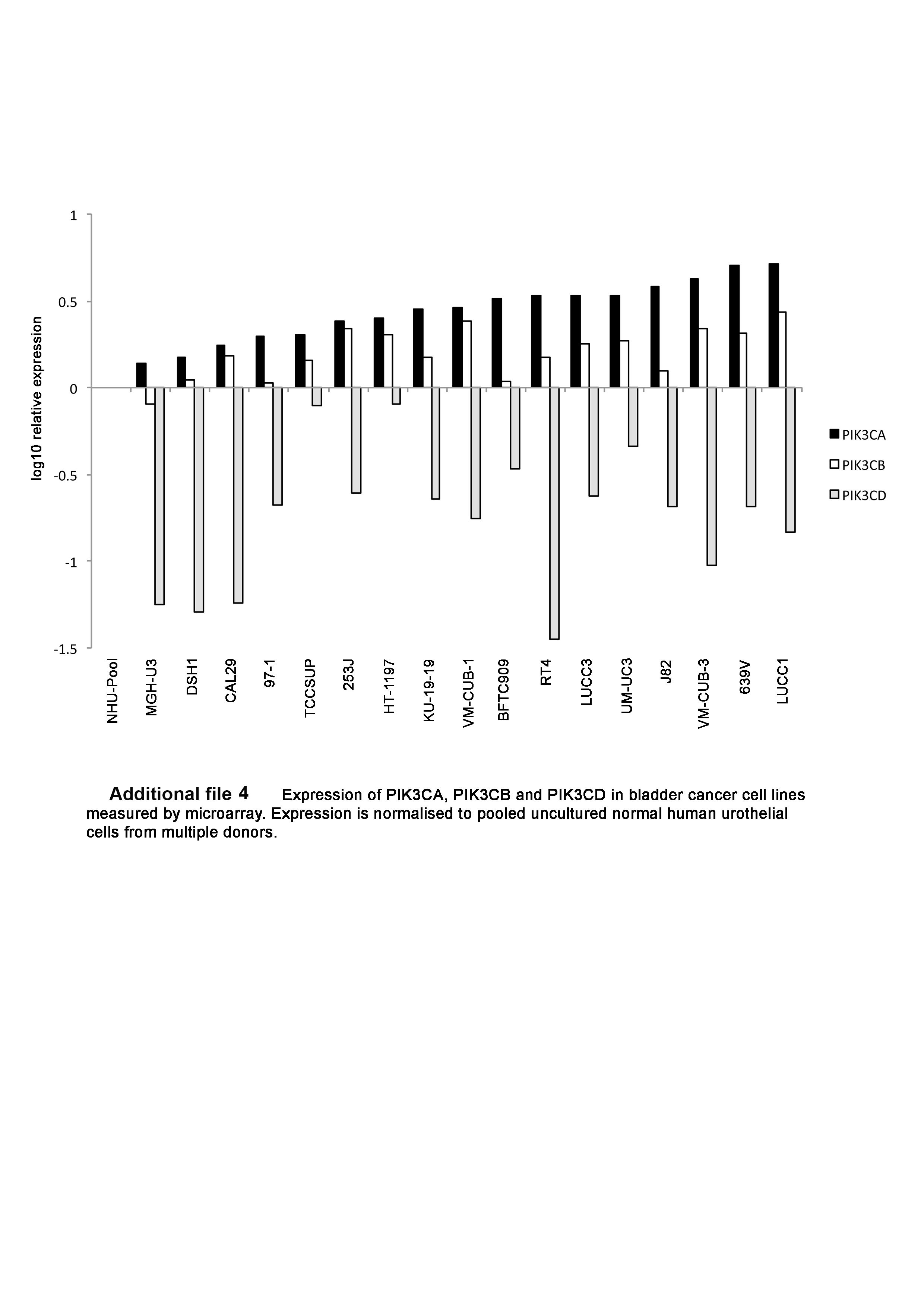

Supplement: Additional file 4: — Expression of PIK3CA, PIK3CB and PIK3CD in bladder cancer cell lines measured by microarray. (TIF 370 kb) [file 12885_2016_2570_MOESM4_ESM.tif]

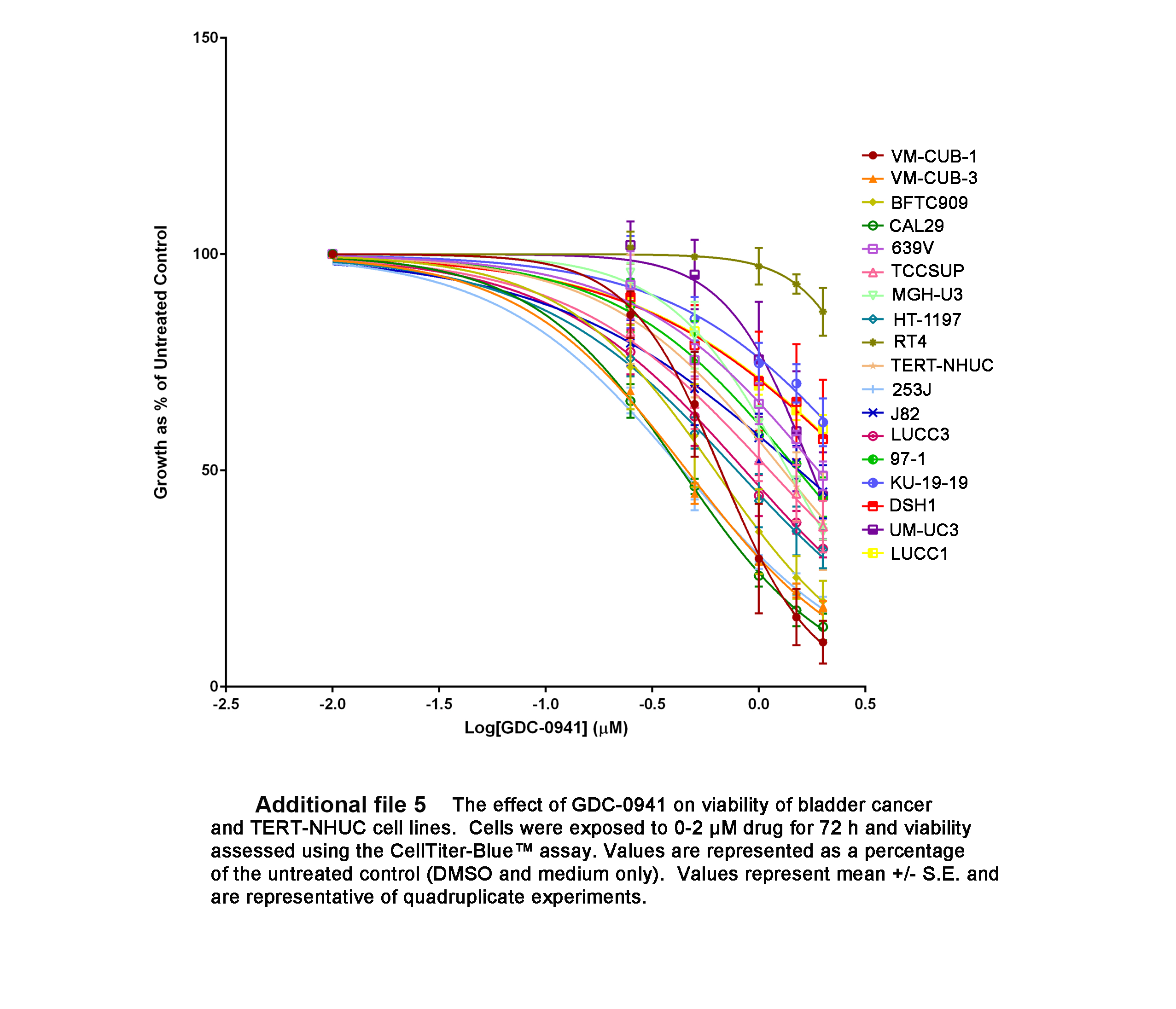

Supplement: Additional file 5: — The effect of GDC-0941 on viability of bladder cancer and TERT-NHUC cell lines. (TIF 701 kb) [file 12885_2016_2570_MOESM5_ESM.tif]
